# Association between urinary arsenic species and vitamin D deficiency: a cross-sectional study in Chinese pregnant women

**DOI:** 10.3389/fpubh.2024.1371920

**Published:** 2024-04-17

**Authors:** Jingran Zhang, Yuxuan Bai, Xi Chen, Shuying Li, Xiangmin Meng, Aifeng Jia, Xueli Yang, Fenglei Huang, Xumei Zhang, Qiang Zhang

**Affiliations:** ^1^Department of Occupational and Environmental Health, School of Public Health, Tianjin Medical University, Tianjin, China; ^2^Key Laboratory of Prevention and Control of Major Diseases in the Population, Ministry of Education, Tianjin Medical University, Tianjin, China; ^3^Department of Endocrinology, Tianjin Xiqing Hospital, Tianjin, China; ^4^Department of Gynecology and Obstetrics, Tianjin Xiqing Hospital, Tianjin, China; ^5^Department of Reproductive Health, Maternal and Child Health Center of Dongchangfu District, Liaocheng, China; ^6^Department of Nutrition and Food Science, School of Public Health, Tianjin Medical University, Tianjin, China

**Keywords:** arsenic species, vitamin D deficiency, pregnancy, BKMR, cross-sectional study

## Abstract

**Background:**

An increasing number of studies suggest that environmental pollution may increase the risk of vitamin D deficiency (VDD). However, less is known about arsenic (As) exposure and VDD, particularly in Chinese pregnant women.

**Objectives:**

This study examines the correlations of different urinary As species with serum 25 (OH) D and VDD prevalence.

**Methods:**

We measured urinary arsenite (As^3+^), arsenate (As^5+^), monomethylarsonic acid (MMA), and dimethylarsinic acid (DMA) levels and serum 25(OH)D_2_, 25(OH)D_3_, 25(OH) D levels in 391 pregnant women in Tianjin, China. The diagnosis of VDD was based on 25(OH) D serum levels. Linear relationship, Logistic regression, and Bayesian kernel machine regression (BKMR) were used to examine the associations between urinary As species and VDD.

**Results:**

Of the 391 pregnant women, 60 received a diagnosis of VDD. Baseline information showed significant differences in As^3+^, DMA, and tAs distribution between pregnant women with and without VDD. Logistic regression showed that As^3+^ was significantly and positively correlated with VDD (OR: 4.65, 95% CI: 1.79, 13.32). Meanwhile, there was a marginally significant positive correlation between tAs and VDD (OR: 4.27, 95% CI: 1.01, 19.59). BKMR revealed positive correlations between As^3+^, MMA and VDD. However, negative correlations were found between As^5+^, DMA and VDD.

**Conclusion:**

According to our study, there were positive correlations between iAs, especially As^3+^, MMA and VDD, but negative correlations between other As species and VDD. Further studies are needed to determine the mechanisms that exist between different As species and VDD.

## 1 Introduction

Vitamin D (VD) plays an essential role in the body’s calcium and phosphorus metabolism, while its deficiency is a widespread nutritional epidemic, affecting approximately one billion individuals worldwide (1, 2). Pregnant women are particularly susceptible to acquiring vitamin D deficiency (VDD), which can lead to hypertension, anemia, and adverse pregnancy outcomes (3, 4). Inadequate exposure to sunlight and a lack of dietary intake of VD are the primary causes of VDD, but environmental pollution, such as exposure to Endocrine Disrupting Chemicals (EDCs) like Polycyclic Aromatic Hydrocarbons (PAHs) may also contribute (5). Furthermore, heavy metals such as Cd, Mn, Pb, and U could affect physiological processes related to sterol metabolism, leading to VDD (6, 7). Currently, there are also some studies suggesting arsenic exposure is associated with VDD (8, 9).

Environmental chemical pollution has become a major global health concern, with arsenic (As) being recognized as one of the most common environmental contaminants responsible for various epidemics (10). Arsenic has been found in the environment in both inorganic and organic forms (11). Inorganic arsenic (iAs) compounds, such as arsenite (As^3+^) and arsenate (As^5+^), are commonly found in certain food sources and drinking water (12, 13). On the other hand, organic As is mainly found in seafood, including arsenobetaine (AsB), arsenosugars, and arsenolipids (14, 15). When As enters the human body through various exposure routes, it is metabolized in the organism by methylation. S-adenosyl methionine (SAM) plays an important physiological role as the sole methyl donor in the methylation reactions that take place in the cell. SAM, on the other hand, is derived from the OCM cycle and is closely linked to folate, vitamin B_12_ and homocysteine (Hcy) (16). Chronic As exposure and its methylation have been proven to be correlated with a series of problems, such as cardiovascular disease, cognitive impairment, hepatic and renal disease, diabetes, and poor pregnancy outcomes (17, 18). Pregnant women, especially those with sensitive conditions, should minimize their exposure to environmental chemicals during pregnancy, particularly As exposure, as it is closely linked to maternal and/or infant health (19).

Currently, there are a number of studies suggesting a possible link between As and VD in the human body. Parvez et al. suggested that there may be unanticipated interactions between As and/or its metabolites and VD (9); additionally, an association between diabetes mellitus, low levels of VD, and As exposure has been observed, although the underlying mechanisms remain unclear (8). We have to make assumptions about these circumstances because there is not much relevant research on this subject. Our study hypothesis is that pregnant women who are exposed to As may have VDD due to altered metabolism; the exact reason may be linked to the combined metabolism of As and VD in the liver. Because As is an endocrine disruptor, it is possible that it affects the way enzymes involved in 25(OH)D production are expressed. As a result, we wanted to find a possible association between prenatal As exposure and the prevalence of VDD in pregnant women. To investigate this relationship, our study examines the relationship between individual As species and both 25(OH)D and VDD by Linear relationship and Logistic regression, and the combined effects of As species on both 25(OH)D and VDD by BKMR. The study aims to identify potential health risks associated with prenatal exposure to environmental As, provide recommendations for maintaining a healthy lifestyle during pregnancy, and establish a scientific basis for evaluating the environmental risk factors contributing to VDD.

## 2 Methods

### 2.1 Study population

The background of this study has been extensively described in the published literature of our previous study (20). A total of 411 pregnant women who received their prenatal care at 24–28 weeks of gestation in Tianjin, China, between October 2017 and January 2018 were enrolled in our study. A systematic questionnaire was employed to gather statistical data on various factors in the population of pregnant women, such as smoking status, alcohol intake, parity, family history of diabetes, gestational diabetes mellitus (GDM), height, current and pre-pregnancy weight, and ethnicity and education. Based on this study, we conducted a follow-up study of this population and measured their serum 25(OH)D_2,_ 25(OH)D_3,_ and 25(OH)D levels. Urine samples and fasting blood were obtained from the maternal population during the assessment of 25(OH)D levels in the serum of pregnant women. After waiting for clot formation, we centrifuged the blood samples and serum was obtained. Samples of urine and serum were aliquoted and subsequently frozen before being sent to Tianjin Medical University for immediate storage at a temperature of-80°C. These samples were kept at this temperature until they were needed for analysis. Of the total sample size of 411 participants, 396 provided sufficient urine and serum samples for As species and VD determination. Women with a history of alcohol consumption and/or smoking were excluded (*n* = 5). Following the conclusive screening process, 391 participants were included in the final analysis.

The Ethics Committee of the Hospital of Metabolic Diseases and the Institute of Endocrinology, Tianjin Medical University, China approved the study protocol. All procedures were conducted in accordance with the Declaration of Helsinki. Prior to their involvement in this study, informed consent was obtained from all individuals.

### 2.2 Determination of urinary as species

The identification of the five forms of As (As^3+^, As^5+^, MMA, DMA, and AsB) in the urine samples was conducted using high-performance liquid chromatography (HPLC) coupled with inductively coupled plasma mass spectrometry (ICP-MS). The HPLC instrument used was the Agilent 1,200, manufactured by Agilent Technologies in California, United States. The ICP-MS instrument used was the Agilent 7500cx, also manufactured by Agilent Technologies in California, United States. The method employed for this determination was based on the procedure described by Wang et al. (21). Briefly, urine samples were allowed to thaw at room temperature for a duration of two hours prior to testing. After vortexing, a volume of 1 mL of urine was subjected to filtration using a polyether sulfone membrane filter with a pore size of 0.22 μm. The filtrate was subsequently transferred to a 1.5 mL autosampler vial in preparation for analysis using High-Performance Liquid Chromatography-Inductively Coupled Plasma Mass Spectrometry (HPLC-ICP-MS). The calibration curves were examined at 0, 0.25, 0.5, 1, 2.5, 5, 10, 25, and 50 μg/L. To assess the performance of the instrument, the standard reference material (with a level of 10 μg/L) was injected at regular intervals into every 20 samples. The recovery range of urine samples spiked with 10 μg/L of As species is 88–105%. The detection limits for As^3+^, MMA, DMA, and AsB were determined to be 0.2 μg/L, whereas the detection limit for As^5+^ was found to be 0.5 μg/L.

### 2.3 Determination of 25(OH)D

The liquid chromatography–tandem mass spectrometry method (WS/T 677–2020, the health industry standard of the People’s Republic of China) was employed to measure 25(OH)D levels in fasting venous blood serum (22). 25(OH)D_2_ and 25(OH)D_3_ are considered to be the 25(OH)D levels. The sample solutions preparation was as follows: 200 μL serum (plasma) sample was taken into a 2 mL centrifuge tube, 20 μL mixed isotope internal standard solution was added, vortexed for 30 s, 400 μL precipitant (methanol + acetonitrile, 1:1) was added, vortexed for 30 s at room temperature, 1.2 mL n-hexane was added, vortexed for 5 min at room temperature, then centrifuged for 5 min at 4°C and centrifugal force >12000 g. The supernatant was pipetted into a 1.5 mL centrifuge tube and blown dry with nitrogen at room temperature. 100 μL of the initial mobile phase was used to redissolve the supernatant, which was then vortexed and shaken for 30 s at room temperature, then centrifuged at 4°C and a centrifugal force of more than 12,000 g for 5 min, and then the supernatant was transferred to the injection vial for analysis by LC-MS/MS. Calibration curves for 5, 10, 25, 50, 125, 250, and 500 μL of 25(OH)D were analyzed. The recoveries for this method ranged from 84.0 to 106.0%. The lower detection limits for 25(OH)D_2_ and 25(OH)D_3_ were determined to be 0.15 ng/mL.

### 2.4 Diagnosis of VDD

According to the screening method for vitamin D deficiency in the population published by the National Health Commission of the People’s Republic of China (NHC) in 2020, individuals can be diagnosed with VDD if their 25(OH)D levels fall below 12 ng/mL in the serum (22).

### 2.5 Statistical analysis

Descriptive statistics were used to compile the demographic and baseline information of the study population. While categorical data were shown as numbers and percentages, non-normal continuous variables were shown as medians (interquartile range, IQR). The Chi-squared test and Fisherʼs exact test were used to assess differences between categorical variables. In this study, nonparametric tests (such as the Mann–Whitney U test) were employed to assess and analyze the disparities in continuous variables between different groups. As^3+^, As^5+^, MMA, DMA are considered as total As (tAs) levels. The skewed distributions of urinary As species levels necessitated the use of the median (IQR) and natural logarithm (ln) transformation to provide the values of all forms of adjusted urinary As^3+^, As^5+^, MMA, DMA, and tAs for subsequent analysis.

**Figure 1:**
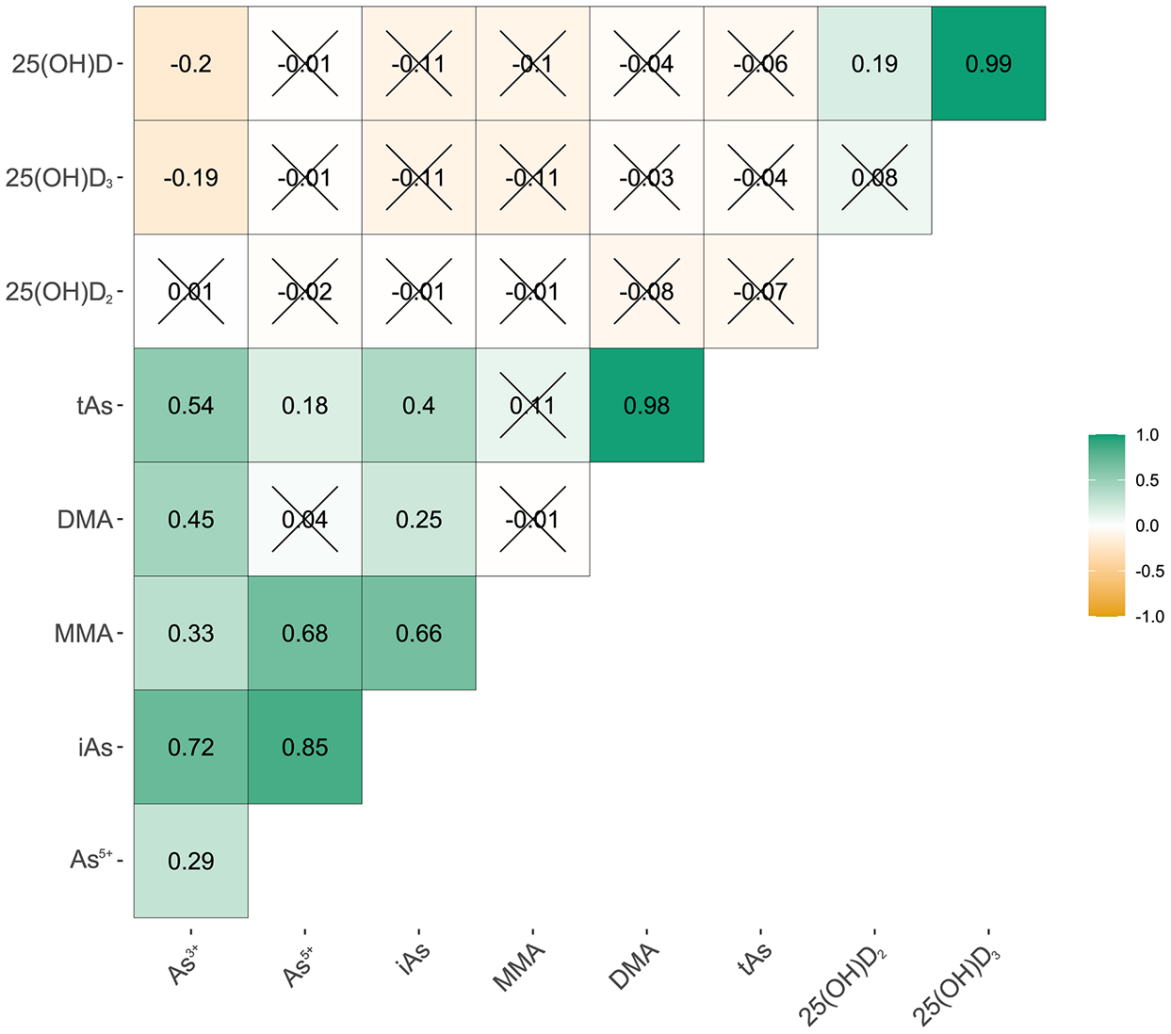
Correlation between arsenic species and different Vitamin D. As^3+^, arsenite; As^5+^, arsenate; MMA, arsenate; DMA, dimethylarsinic acid; iAs, inorganic arsenic; tAs, total urinary As; 25(OH)D_2_, 25-Hydroxyvitamin D_2_; 25(OH)D_3_, 25-Hydroxyvitamin D_3_; 25(OH)D, 25-Hydroxyvitamin D (“×”*p* ≥ 0.05).

To evaluate the degree of correlation between As species, total arsenic (tAs), and 25(OH)D, Spearman’s rank correlation coefficients were calculated and are shown in Figure 1. Linear relationship and Logistic regression are used to evaluate the relationship between specific As species and tAs with VD and VDD. First, to explore the association between arsenic species, tAs variables, and 25(OH)D_2_, 25(OH)D_3_, 25(OH)D variables, linear regression analysis was employed. The regression coefficient *β* and its corresponding 95% confidence interval (95% CI) will serve as the foundation for evaluation. The association between As exposure and VDD was then evaluated using a logistic regression analysis, which was visualized by odds ratios (ORs) and 95% CIs. As exposure was assessed both as a continuous variable and a categorical variable (specified by tertiles). The binary outcome variable was used in the logistic regression model. In Linear relationship and Logistic regression, it is common practice to account for the relevant confounding variables. To account for potential confounding factors, two models were employed. Model 1 was modified to account for potential confounding variables related to the mother, including age, ethnicity, education level, and pre-pregnancy BMI. All the variables in Model 1 were included in Model 2, along with extra changes in serum markers of OCM like folate, vitamin B_12_, and Hcy. The study also looked at concurrent urinary AsB and how GDM affected the outcome.

The BKMR method was used to evaluate the correlation between mixtures of As species and 25(OH)D levels and VDD status to evaluate the potential non-linear and non-additive correlation of As species with serum 25(OH)D and VDD. BKMR uses previously described Gaussian kernel functions (23). A combination of Bayesian and statistical learning methods simulated the individual and joint effects of mixed exposures. In the present study, separate BKMR models were created for each form of VDD based on the following model:

$Y_{i}=h\;\left({\mathrm{As}}_{i}3+\mathrm{,As}i5+\mathrm{,MMA}i\;\mathrm{,DMA}i\right)+\beta^{T}Z_{i}+e_{i}$

where Y_i_ represents a continuous result related to VD levels (specifically, 25(OH)D_2_, 25(OH)D_3_, and 25(OH)D) or a binary outcome related to VDD. h() is the exposure–response function based on nonlinearity and/or interaction among As species, Z_i_ is a vector that allows for confounders adjustment (β is the corresponding vector of coefficients), e_i_ is a random error term. Arsenic species were scaled for BKMR analyses. Model adjustment included the incorporation of maternal and dietary confounders, including age, ethnicity, education level, pre-pregnancy BMI, folate, vitamin B_12_, and Hcy, in addition to urinary AsB and GDM status. Model parameters were obtained using a Markov chain Monte Carlo sampler, specifically within the bkmr R package, with a total of 10,000 iterations. The results of these models were condensed and then utilized to establish the exposure-response correlations between each As species and the 25(OH)D levels, and VDD, while keeping all other species at their median values. In the present study, we wanted to evaluate the correlation between the increasing interquartile range of each As species and the 25(OH)D level content, in addition to the prevalence of VDD. This analysis was conducted while keeping all other species at a fixed percentile or at either the 25th, 50th, or 75th percentile.

The statistical analyses were conducted using R (version 4.0.2; R Project for Statistical Computing). The BKMR analysis was conducted using the “bkmr” package in R, specifically version 0.2.0. In the current study, all significance values were set at 0.05.

## 3 Results

### 3.1 Baseline characteristics

Table 1 shows the baseline information for the study population. Of the total sample size of 391 pregnant women, 60 were found to be diagnosed with VDD. The median values of pre-pregnancy BMI and age within the study were 22.7 kg/m^2^ and 29.0 years, respectively. The majority of the study participants were identified as belonging to the Han ethnic group, accounting for 94.4% of the total sample. Among the entire cohort, a total of 32 individuals (8.2%) indicated the presence of a familial predisposition to diabetes. Meanwhile, we categorized individuals based on the presence or absence of VDD and examined the characteristics associated with each group. The findings indicated a significant difference in As^3+^ levels between those with VDD (0.9 μg/L) and those with normal VD levels (0.8 μg/L). When comparing the data, it was observed that the levels of DMA (10.6 μg/L vs. 14.3 μg/L) and tAs (13.8 μg/L vs. 17.3 μg/L) in their bodies increased with significant differences.

**Table 1:** Baseline information of the study population by Vitamin D deficiency status.

Maternal characteristics	All participants (*n* = 391)	Non-vitamin D deficient (*n* = 331)	Vitamin D deficient (*n* = 60)	*p* value
Age (years)	29.0 (26.0, 32.0)	29.0 (27.0, 32.5)	27.5 (25.0, 32.0)	0.025
Pre-pregnancy BMI (kg/m^2^)	22.7 (20.6, 25.4)	22.8 (20.8, 25.4)	22.3 (19.5, 25.4)	0.310
Ethnicity, n (%)				0.759
Minority Nationality	22 (5.6)	18 (5.4)	4 (6.7)	
Han nationality	369 (94.4)	313 (94.6)	56 (93.3)	
Education (years), n (%)				0.529
<=12	151 (38.6)	158 (47.7)	38 (63.3)	
>12	240 (61.4)	173 (52.3)	22 (36.7)	
Parity, n (%)				0.025
Nulliparous	196 (50.1)	104 (54.5)	92 (46.0)	
Multiparous	195 (49.9)	87 (45.5)	108 (54.0)	
Family history of diabetes, n (%)				0.199
No	359 (91.8)	301 (90.9)	58 (96.7)	
Yes	32 (8.2)	30 (9.1)	2 (3.3)	
GDM, n (%)				<0.001
non-GDM	304 (77.7)	247 (74.6)	57 (95.0)	
GDM	87 (22.3)	84 (25.4)	3 (5.0)	
OCM nutrients				
Folate (ng/mL)	8.9 (6.2, 14.3)	9.5 (6.6, 14.9)	6.7 (4.8, 9.5)	<0.001
Vitamin B_12_ (pg/mL)	271.0 (212.0, 336.5)	272.0 (215.0, 345.5)	256.0 (183.0, 299.8)	0.023
Hcy (μmol/L)	5.1 (4.53, 6.1)	5.05 (4.5, 6.1)	5.10 (4.6, 6.1)	0.647
As species (μg/L)				
As^3+^	0.8 (0.5, 1.2)	0.8 (0.4, 1.1)	0.9 (0.6, 1.5)	0.006
As^5+^	2.0 (1.6, 2.5)	2.0 (1.6, 2.5)	2.1 (1.6, 2.4)	0.898
iAs	2.76 (2.19, 3.48)	2.7 (2.2, 3.4)	3.0 (2.3, 3.8)	0.141
MMA	0.15 (0.12, 0.21)	0.2 (0.1, 0.2)	0.2 (0.1, 0.2)	0.130
DMA	11.0 (7.5, 16.4)	10.6 (7.4, 15.6)	14.3 (9.4, 19.8)	0.020
tAs	14.1 (10.4, 19.8)	13.8 (10.3, 19.3)	17.3 (12., 23.8)	0.016
AsB	6.27 (3.3, 13.5)	6.32 (3.2, 13.8)	5.62 (3.7, 11.6)	0.709

Non-normally distributed variables are presented as medians (IQR). Categorical variables are presented as numbers (percentages).

### 3.2 Associations between urinary as species and serum 25(OH)D

Figure 1 shows how the As^3+^, As^5+^, MMA, DMA, and tAs are related to serum 25(OH)D_2_, 25(OH)D_3_, and 25(OH)D. The Spearman’s correlation coefficient between As^3+^ and 25(OH)D_3_ was −0.19, while the correlation coefficient with 25(OH)D was −0.20. The statistical significance of the above indicators was significant. There were no significant correlations between other indicators.

A significant negative correlation was observed between As^3+^ and 25(OH)D_3_, as well as 25(OH)D, in the crude model, as shown in Table 2. This was true even when other factors like age, race, education, pre-pregnancy BMI, serum folate, vitamin B_12_, Hcy, urinary AsB, and GDM were taken into account. A one-unit increase in the natural logarithm (ln) of As^3+^ was linked to a drop of 6.24 ng/mL in 25(OH)D_3_ and a drop of 6.45 ng/mL in 25(OH)D in Model 2. In addition, a negative correlation was identified between iAs and 25(OH)D_3_ in the crude model and Model 2. This correlation between iAs and 25(OH)D also exists in the crude model, Model 1 and Model 2. In Model 1, MMA showed a negative correlation with 25(OH)D_3_ and 25(OH)D. DMA was negatively correlated with 25(OH)D_2_ in each model. Also, tAs showed a negative correlation with 25(OH)D_2_ in Model 1. There was no statistically significant correlation identified between As^5+^ and all forms of 25(OH)D, either before or after adjusting for various confounders.

**Table 2:** Linear relationship between arsenic species and different forms of 25(OH)D.

Arsenic (μg/L)	25(OH)D_2_		25(OH)D_3_		25(OH)D	
	*β* (95%CI)	*p* value	*β* (95%CI)	*p* value	*β* (95%CI)	*p* value
Per unit increase in ln (As^3+^)						
Crude model	−0.15 (−0.54, 0.25)	0.461	−5.35 (−8.64, −2.07)	0.001	−5.50 (−8.81, −2.20)	0.001
Model1^a^	−0.17 (−0.57, 0.22)	0.390	−4.99 (−8.26, −1.72)	0.003	−5.16 (−8.45, −1.87)	0.002
Model2^b^	−0.22 (−0.61, 0.18)	0.284	−6.24 (−9.07, −3.40)	<0.001	−6.45 (−9.29, −3.61)	<0.001
Per unit increase in ln (As^5+^)						
Crude model	0.08 (−0.63, 0.80)	0.823	−1.72 (−7.76,4.32)	0.577	−1.64 (−7.72,4.45)	0.597
Model1^a^	0.09 (−0.63, 0.80)	0.816	−2.18 (−8.18,3.82)	0.476	−2.09 (−8.13,3.94)	0.496
Model2^b^	0.16 (−0.57, 0.88)	0.668	0.13 (−5.19,5.46)	0.961	0.29 (−5.06,5,64)	0.915
Per unit increase in ln (iAs)						
Crude model	−0.03 (−0.14, 0.08)	0.593	−0.93 (−1.85, −0.01)	0.047	−0.96 (−1.89, −0.04)	0.041
Model1^a^	−0.04 (−0.14, 0.07)	0.529	−0.91 (−1.82,0.01)	0.051	−0.94 (−1.86, −0.02)	0.044
Model2^b^	−0.02 (−0.13, 0.08)	0.659	−0.83 (−1.63, −0.03)	0.041	−0.86 (−1.66, −0.06)	0.036
Per unit increase in ln (MMA)						
Crude model	0.12 (−0.48, 0.72)	0.693	−4.60 (−9.67,0.47)	0.075	−4.48 (−9.58,0.62)	0.085
Model1^a^	0.09 (−0.52, 0.70)	0.773	−5.45 (−10.50, −0.40)	0.035	−5.36 (−10.45, −0.27)	0.039
Model2^b^	0.14 (−0.47, 0.75)	0.644	−2.48 (−6.96,2.00)	0.278	−2.33 (−6.83,2.17)	0.308
Per unit increase in ln (DMA)						
Crude model	−0.52 (−1.04, −0.10)	0.047	−0.99 (−5.38,3.40)	0.658	−0.88 (−5.23,3.46)	0.690
Model1^a^	−0.55 (−1.07, −0.03)	0.038	−0.46 (−4.81,3.89)	0.836	−1.01 (−5.39,3.37)	0.651
Model2^b^	−0.54 (−1.06, −0.01)	0.047	−1.92 (−5.81,1.98)	0.333	−2.45 (−6.36,1.45)	0.218
Per unit increase in ln (tAs)						
Crude model	−0.02 (−0.03, 0.01)	0.064	−0.09 (−0.23,0.05)	0.219	−0.11 (−0.25,0.04)	0.150
Model1^a^	−0.02 (−0.03, −0.01)	0.044	−0.07 (−0.21,0.07)	0.311	−0.09 (−0.23,0.05)	0.213
Model2^b^	−0.02 (−0.03, 0.10)	0.069	−0.10 (−0.23,0.03)	0.119	−0.12 (−0.24,0.01)	0.072

^a^Model 1: adjusted for age, ethnicity, education, and pre-pregnancy BMI. ^b^Model 2: adjusted for variables in model 1, and serum folate, vitamin B_12_, Hcy, urinary AsB, and GDM.

### 3.3 Associations between as species and VDD in logistic regression

The relationships between As species and tAs and the presence of VDD are presented in Table 3. In the tertiles of As^3+^, high tertiles were statistically significantly associated with VDD in each model, and the OR and corresponding 95% CIs were as follows: 2.36 (1.16, 4.80), 2.24 (1.08, 4.62) and 2.82 (1.31, 6.10). Additionally, there was a statistically significant correlation between the tertiles of DMA, tAs and VDD in the partial circumstance. Also, the OR (95% CI) for high tertiles in Model 2, corresponding to DMA and tAs, was 3.29 (1.51, 7.16) and 2.89 (1.36, 6.16), respectively.

**Table 3:** Logistic regression of arsenic species and vitamin D deficiency.

Arsenic (μg/L)^a^	VDD (n)	Non-VDD (n)	OR (95%CI)^b^	*p* value	OR (95%CI)^c^	*p* value	OR (95%CI)^d^	value
As^3+^ tertiles								
Low (<0.58)	13	119	1.0		1.0		1.0	
Medium (0.59–1.00)	20	109	1.66 (0.79, 3.51)	0.180	1.50 (0.71, 3.20)	0.289	1.88 (0.85, 4.17)	0.121
High (≥1.00)	27	104	2.36 (1.16, 4.80)	0.018	2.24 (1.08, 4.62)	0.029	2.82 (1.31, 6.10)	0.008
Per unit increase in ln (As^3+^)	60	331	3.40 (1.41, 8.85)	0.009	3.25 (1.31, 8.66)	0.014	4.65 (1.79, 13.32)	0.003
As^5+^ tertiles								
Low (<1.73)	22	109	1.0		1.0		1.0	
Medium (1.73–2.31)	16	114	0.70 (0.35, 1.39)	0.306	0.67 (0.33, 1.35)	0.259	0.70 (0.33, 1.48)	0.356
High (≥2.31)	22	108	1.01 (0.53, 1.93)	0.978	1.09 (0.56, 2.10)	0.800	0.93 (0.46, 1.87)	0.835
Per unit increase in ln (As^5+^)	60	331	1.03 (0.25, 4.72)	0.966	1.25 (0.30, 5.77)	0.767	0.74 (0.16, 3.76)	0.705
iAs tertiles								
Low (<2.41)	16	115	1.0		1.0		1.0	
Medium (2.41–3.26)	18	112	1.16 (0.56, 2.38)	0.695	1.12 (0.54, 2.31)	0.767	1.24 (0.58, 2.67)	0.585
High (≥3.26)	26	104	1.80 (0.91, 3.54)	0.089	1.81 (0.91, 3.60)	0.092	1.92 (0.92, 4.01)	0.080
Per unit increase in ln (iAs)	60	331	3.17 (0.64, 16.87)	0.167	3.35 (0.66, 18.50)	0.156	3.22 (0.59, 19.49)	0.191
MMA tertiles								
Low (<0.13)	16	113	1.0		1.0		1.0	
Medium (0.13–0.18)	19	114	1.18 (0.58, 2.40)	0.654	1.17 (0.56, 2.41)	0.672	1.13 (0.53, 2.42)	0.757
High (≥0.18)	25	104	1.70 (0.86, 3.36)	0.128	1.86 (0.93, 3.71)	0.080	1.57 (0.76, 3.26)	0.226
Per unit increase in ln (MMA)	60	331	2.02 (0.61, 6.43)	0.242	2.58 (0.76, 8.45)	0.121	1.80 (0.51, 6.28)	0.356
DMA tertiles								
Low (<8.97)	13	117	1.0		1.0		1.0	
Medium (8.97–14.28)	17	114	1.34 (0.62, 2.89)	0.453	1.29 (0.59, 2.79)	0.525	1.42 (0.63, 3.19)	0.399
High (≥14.28)	30	100	2.70 (1.34, 5.45)	0.006	2.61 (1.28, 5.34)	0.009	3.29 (1.51, 7.16)	0.003
Per unit increase in ln (DMA)	60	331	2.16 (0.73, 6.79)	0.177	2.02 (0.65, 6.58)	0.234	2.54 (0.80, 8.75)	0.127
tAs tertiles								
Low (<1.59)	14	117	1.0		1.0		1.0	
Medium (1.59–1.97)	15	115	1.09 (0.50, 2.36)	0.828	1.07 (0.49, 2.33)	0.870	0.98 (0.44, 2.22)	0.971
High (≥2.49)	31	99	2.62 (1.32, 5.19)	0.006	2.53 (1.26, 5.08)	0.009	2.89 (1.36, 6.16)	0.006
Per unit increase in ln (tAs)	60	331	3.45 (0.91, 13.62)	0.073	3.32 (0.83, 13.74)	0.093	4.27 (1.01, 19.59)	0.055

^a^Arsenic exposure was evaluated as a categorical variable (defined by tertiles) and as a continuous variable. ^b^Crude model. ^c^Model 1: adjusted for age, ethnicity, education, and pre-pregnancy BMI. ^d^Model 2: adjusted for variables in model 1, and serum folate, vitamin B_12_, Hcy, urinary AsB, and GDM.

### 3.4 Associations between as species and VDD in BKMR

**Figure 2:**
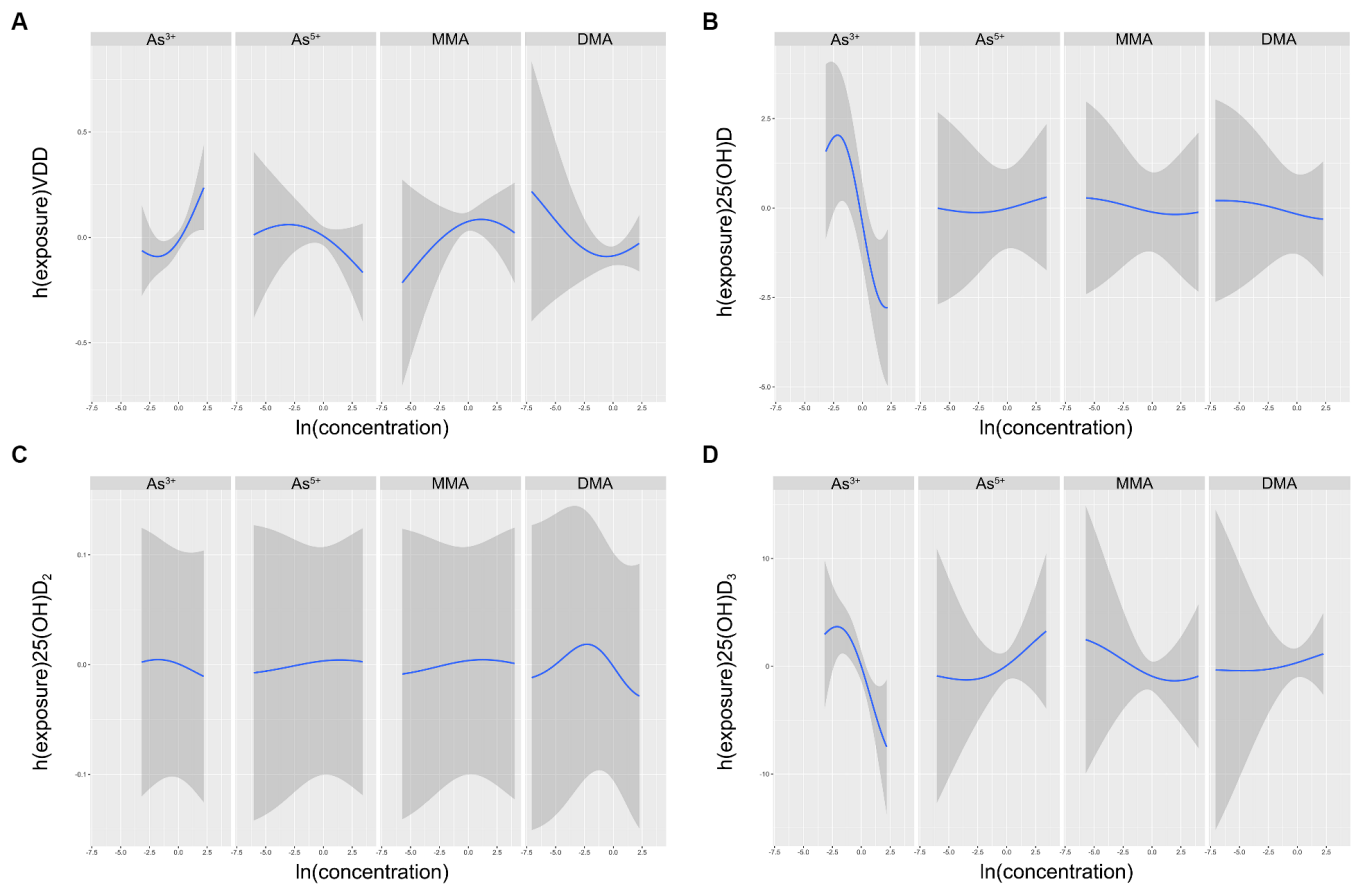
Univariate exposure-response functions (95% CI) between different arsenic species and VDD **(A)**, 25(OH)D **(B)**, 25(OH)D_2_ **(C)**, 25(OH)D_3_ **(D)** with the other species fixed at the median. The results were assessed using the BKMR model adjusted for age, ethnicity, education, pre-pregnancy BMI, serum folate, vitamin B_12_ and Hcy, as well as urinary MMA and GDM.

**Figure 3:**
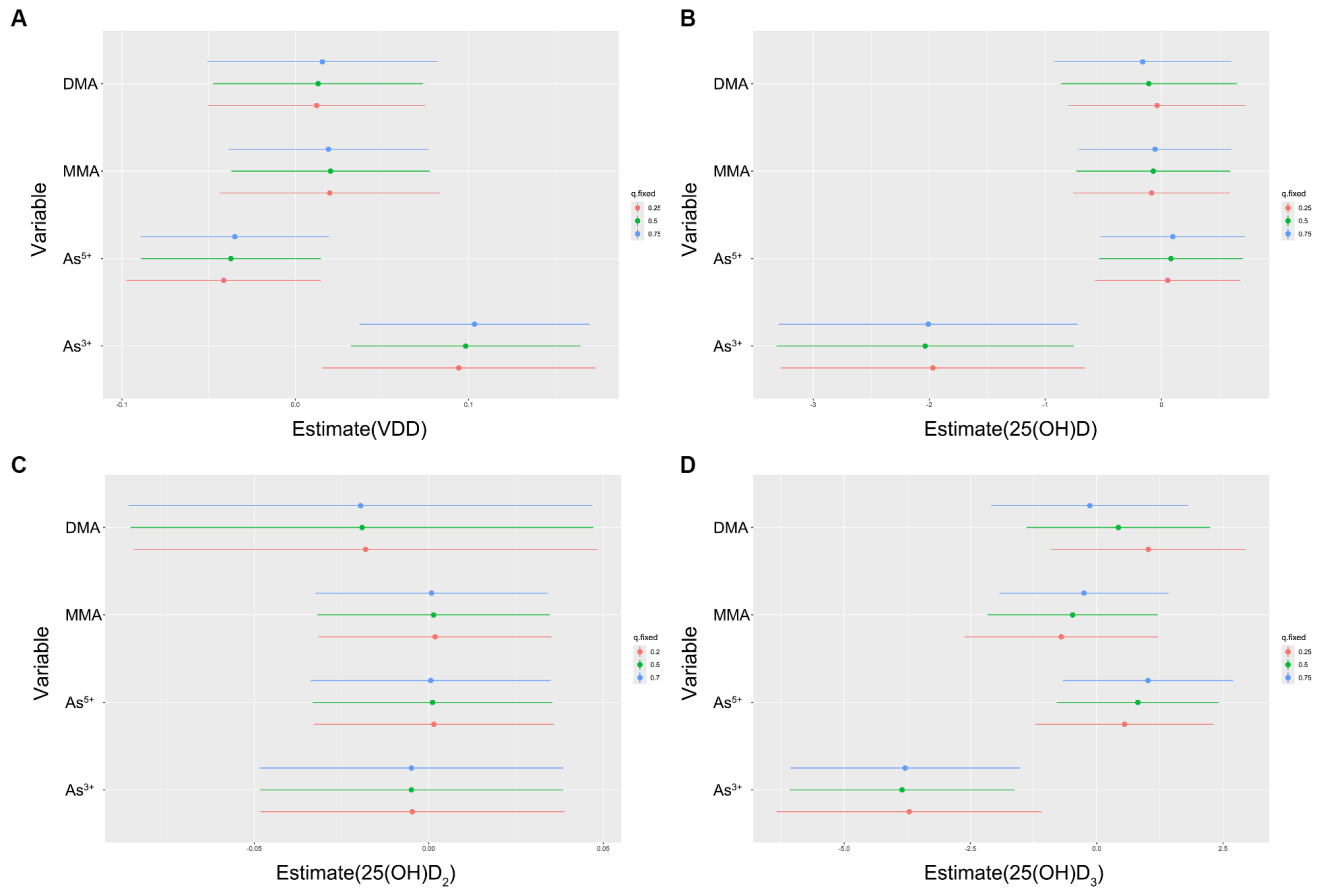
Individual pollutant associations (estimates and 95% CIs) between arsenic species and VDD **(A)**, 25(OH)D **(B)**, 25(OH)D_2_ **(C)**, 25(OH)D_3_ **(D)**. This graph compares serum vitamin D levels or VDD when a single arsenic species was at the 75th vs. 25th percentile, with all other arsenic species fixed at either the 25th, 50th, or 75th percentile. Results were evaluated using the BKMR model adjusted for age, ethnicity, education, pre-pregnancy BMI, serum folate, vitamin B_12_ and Hcy, in addition to urinary MMA and GDM.

Four As species exposure-response functions, with the three other As species fixed at their respective median values, showed that the relationship between As^3+^ and VDD increased and are displayed in Figure 2. Conversely, the relationship between As^3+^ and 25(OH)D_2_, 25(OH)D_3_, and 25(OH)D decreased in Figure 3. Nevertheless, the variable As^5+^ exhibited a significant decrease in its relationship with VDD while showing an increasing trend in its relationship with 25(OH)D_2_, 25(OH)D_3_ and 25(OH)D. The study also showed a negative correlation between MMA and 25(OH)D and 25(OH)D_3_, while showing a favorable correlation with VDD and 25(OH)D_2_. DMA had an overall negative correlation with 25(OH)D, while showing a positive correlation with 25(OH)D_3_.

When a single As species increased from the 25th to the 75th percentile, and other species remained at different percentiles (25th, 50th, or 75th percentile), the results indicated a strong positive correlation between As^3+^ and VDD. In comparison, the As^5+^ had significantly different outcomes. Both MMA and DMA demonstrated a significant positive correlation with VDD, while showing a different correlation with 25(OH)D_2_, 25(OH)D_3_ and VDD.

## 4 Discussion

In this study, we investigated the association between urinary As species and VDD. We found that demographic characteristics such as age, parity and family history of diabetes, OCM nutrients and other confounding factors may affect As metabolism (24–26). Although we made some adjustments for these confounders, the relationship between As^3+^ and VDD did not change significantly. Thus, our findings indicate that subjects with higher levels of As^3+^ are more likely to develop VDD, although the cross-sectional study can only provide weak evidence of causality.

The results of this investigation of different forms of As species are crucial for the assessment of epidemiological findings and other As-related health effects, especially for pregnant women, a group that is vulnerable to As. One existing study provided a summary indicating that OCM nutrients have been shown to increase the ability of the general population to methylate As, but the impact on pregnant women remains uncertain, and the findings are inconclusive (16). This is therefore an area for future research. Additionally, based on our study of the joint effect of urinary As species and serum OCM nutrients on gestational diabetes mellitus (GDM) and Lee’s study, we recognized that the GDM status of pregnant women may have some impact on their VD status (8, 27). Therefore, we used them as correction factors and included them in the adjusted models, and further data analysis was carried out.

Turning our focus to another essential nutrient, VD, we encountered a prominent figure in the realm of fat-soluble secosterols. VD consists of two forms, including VD_2_ (ergocalciferol) and VD_3_ (cholecalciferol), which are classified as physiologically inactive hormone precursors (28). Both of these could be obtained from external sources. The former is produced in plants and fungi when ergosterol is exposed to UVB light, whereas the latter is produced in the skin as a major and endogenous source and is found in dietary sources. In conclusion, VD can be obtained primarily through sun exposure, which serves as the primary source, or through dietary intake, which serves as a supplemental source (29). Therefore, any conditions that have the potential to limit sun exposure, whether on a periodic or local basis, may impact the synthesis of VD. Moreover, there are additional environmental elements that may disrupt the VD endocrine system (VDES) and lead to VD insufficiency or even deficiency. In recent years, strong evidence has emerged linking VDD to exposure to environmental contaminants, particularly EDCs (22).

According to the United States Environmental Protection Agency (EPA), an EDC is any substance that enters the bloodstream and interferes with the synthesis, secretion, transport, metabolism, binding activity, or elimination of naturally occurring hormones. These hormones are essential for maintaining homeostasis, promoting reproduction, and assisting in the stages of development (30). Pesticides, metals, bisphenol A (BPA), phthalates, polycyclic aromatic hydrocarbons (PAHs), and polyhalogenated compounds are some of the EDCs that pose the greatest risk to human health (31). As a result, As falls under the EDC classification. Previous studies have shown the effect of EDCs on thyroid hormone (TH) levels and steroid hormone biosynthetic pathways (32, 33). It is likely that EDCs affect VDES because of the chemical structure and biological functions of VD, which are similar to those of steroid hormones, and because its nuclear receptor is a member of the same protein superfamily as thyroid hormone and steroid hormone receptors (34). By using As as a typical EDC in our study, it is reasonable and feasible to look into the association between As exposure and VDD.

We found that participants with VDD had higher As^3+^, DMA, and tAs levels than those without vitamin D deficiency, both of which were statistically significant (Table 1). This is an interesting point, and to explore the reasons behind it, we used GLMs and BKMR for more in-depth statistical analysis. The data suggested a linear negative correlation between As^3+^, iAs and 25(OH)D_3_, as well as between As^3+^, iAs and 25(OH)D. Additionally, a linear negative correlation was found between DMA and 25(OH)D_2_ (Table 2). Logistic regression analysis suggested that exposure to As^3+^ may potentially increase the risk of VDD in all models (OR = 4.65, *p* = 0.003). Meanwhile, exposure to tAs may also have a similar result to VDD, but there is a marginal correlation (OR = 4.27, *p* = 0.055; Table 3). Univariate exposure-response functions from the BKMR model indicated positive correlations between As^3+^, MMA and VDD, but negative correlations between As^5+^, DMA and VDD respectively, as depicted in Figure 2. Hence, it was seen that there was a clear association between As exposure and VDD. It was certain that pregnant women who had elevated levels of As^3+^ had a higher risk of being diagnosed with VDD. The tAs levels when looking at the difference between people with normal VD levels and those with VDD at the start of the study could be due to differences in how different forms of As are distributed in the urine. It is important to acknowledge that in our study, the tAs level was determined by calculating the sum of several As species present in the urine, which is different from using direct measurement to determine the tAs levels in the biosamples.

The potential etiology of VDD in the study population as a result of environmental As exposure is of interest. The metabolic pathways of VD involve three essential enzymatic reactions, namely 25-hydroxylation, 1α-hydroxylation, and 24-hydroxylation. These processes are facilitated by various enzymes called cytochrome P450 mixed-function oxidases, which can be located in different cellular compartments, including the endoplasmic reticulum (ER) or mitochondria. Some examples of these enzymes are CYP2R1, which is located in the endoplasmic reticulum, and CYP27A1, CYP27B1, and CYP24A1, which are located in the mitochondria. The 25-hydroxylation reactions take place within the hepatic system and are aided by many cytochrome P450 enzymes (CYPs) that possess the ability of 25-hydroxylase activity. The formation of 25(OH)D occurs as a consequence of these enzymatic pathways (35, 36). This theory proposes that inhibition of the expression and activities of CYPs by EDCs is the primary mechanism by which As exposure may result in a reduction of serum 25(OH)D levels in the liver, and contribute to the development of VDD. This suggestion provides a valuable opportunity for future research to elucidate the underlying mechanism of how As exposure contributes to the development of VDD.

An important quality of this study is its detailed evaluation of the correlation between different forms of As exposure and different categories of 25(OH)D levels within a specific demographic, namely pregnant women. Our study not only measured different species of As separately but also estimated the combined effect of As species on VDD. Thus, our study provides a scientific basis for research on nutrient deficiencies caused by environmental pollutants. Despite these strengths, there are some limitations to our study. The temporal relationships between As exposure and VDD could not be determined because of the cross-sectional approach used in this study. Follow-up studies are needed for prospective cohort studies to distinguish causality. Second, our present study remained at the indicator level of VDD, and the mechanism of As exposure and VDD is still unclear. There is a gap in the global research on this mechanism, so this also needs to be further investigated by us. Our hypothesis was that the human liver is the site where As affects 25(OH)D levels. This is based on the known processes of methylation metabolism and VD 25-hydroxylation has occurred in the liver. This observation served as a catalyst to advance our work.

## 5 Conclusion

The goal of the present study was to examine the association between different urinary As species and VDD in Chinese pregnant women. Different statistical approaches, Linear relationship, Logistic regression, and BKMR, were employed in our study. Despite the lack of a statistically significant correlation between tAs and VDD, our analysis revealed a significant positive correlation between As^3+^ and VDD. On the other hand, the BKMR model revealed a positive relationship between MMA and VDD, and negative relationships between As^5+^, DMA and VDD. Based on our findings, we believe that pregnant women should avoid iAs exposure, especially As^3+^. The nutritional status of pregnant women is closely related to fetal development. Individuals who are at a higher risk for VDD should consistently monitor their dietary indicators to mitigate the potential negative consequences during pregnancy. In future research endeavors, it will be vital to conduct additional investigations on the effect of different forms of As species on the correlation between different forms of 25(OH)D and to gain a more comprehensive understanding of the mechanisms behind As exposure and VDD.

## Data availability statement

The datasets used and/or analysed during the current study are available from the corresponding author on reasonable request.

## Ethics statement

The studies involving humans were approved by the Ethics Committee of the Hospital of Metabolic Diseases and the Institute of Endocrinology, Tianjin Medical University, China. The studies were conducted in accordance with the local legislation and institutional requirements. Written informed consent for participation in this study was provided by the participants’ legal guardians/next of kin. Written informed consent was obtained from the individual(s) for the publication of any potentially identifiable images or data included in this article.

## Author contributions

JZ: Formal analysis, Visualization, Writing – original draft. YB: Investigation, Writing – original draft. XC: Data curation, Investigation, Writing – original draft. SL: Data curation, Investigation, Writing – review & editing. XM: Investigation, Writing – review & editing. AJ: Investigation, Project administration, Writing – review & editing. XY: Methodology, Resources, Software, Writing – review & editing. FH: Investigation, Methodology, Writing – review & editing. XZ: Funding acquisition, Writing – review & editing. QZ: Funding acquisition, Writing – review & editing.
