# Supplementary material for: Association between urinary arsenic species and vitamin D deficiency: a cross-sectional study in Chinese pregnant women
Source: Front Public Health. 2024 Apr 17;12:1371920. doi: 10.3389/fpubh.2024.1371920 (PMC11062242; doi:10.3389/fpubh.2024.1371920)

**Table S1.** The detection of arsenic species and vitamin D

| Arsenic | As^3+^ | As^5+^ | MMA | DMA | AsB | 25(OH)D_2_ | 25(OH)D_3_ | 25(OH)D |
| --- | --- | --- | --- | --- | --- | --- | --- | --- |
| detection, n (%) | 341 (87.21%) | 388 (99.23%) | 37 (9.46%) | 390 (99.74%) | 388 (99.23%) | 391(100%) | 391(100%) | 391(100%) |

**Figure S1.** Flowchart of participant selection.


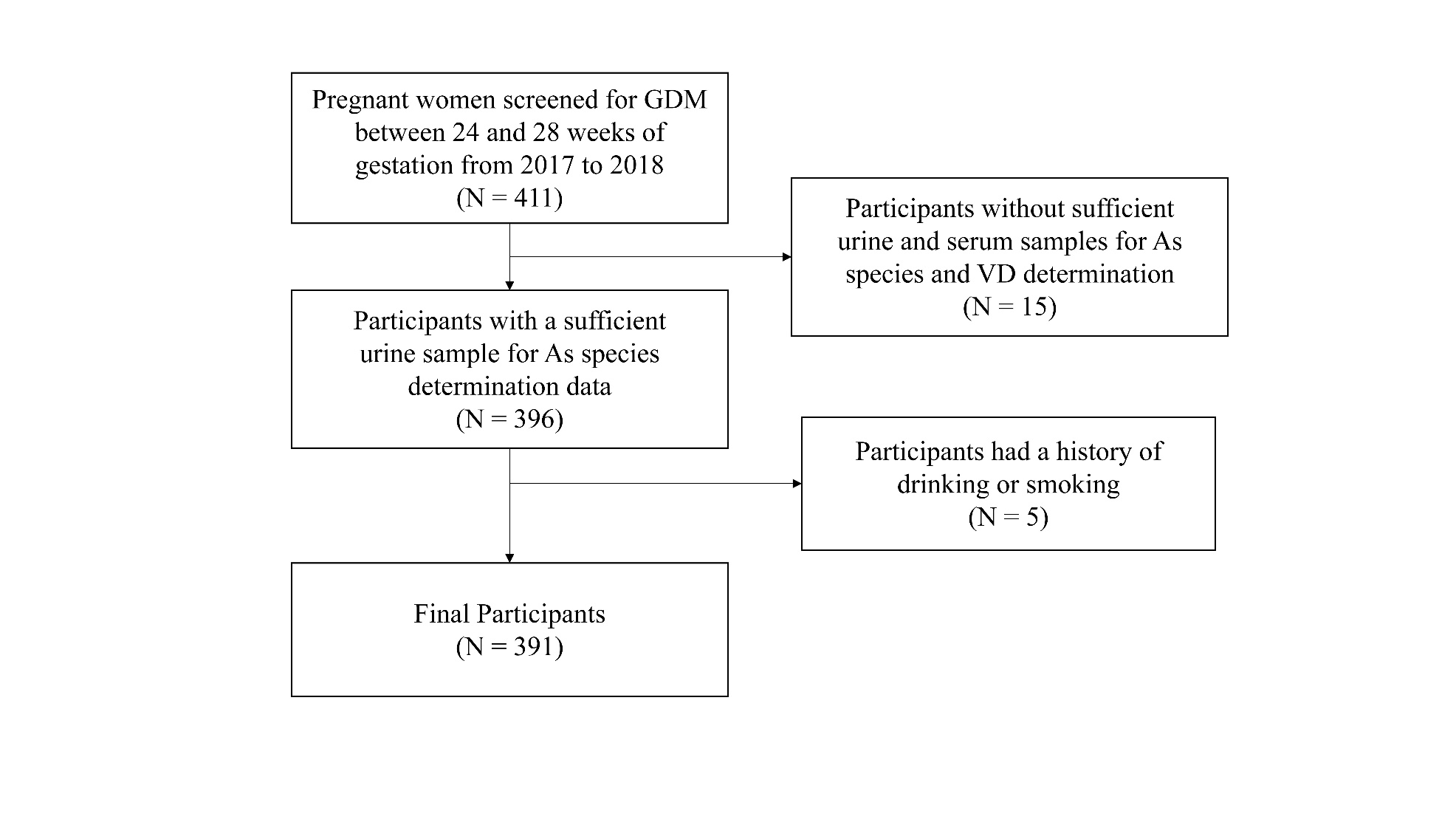

Supplement: Supplementary file 1 [file Table_1.docx]
